# Triggering and recovery of earthquake accelerated landslides in Central Italy revealed by satellite radar observations

**DOI:** 10.1038/s41467-022-35035-5

**Published:** 2022-11-29

**Authors:** Chuang Song, Chen Yu, Zhenhong Li, Stefano Utili, Paolo Frattini, Giovanni Crosta, Jianbing Peng

**Affiliations:** 1 College of Geological Engineering and Geomatics, Chang’an University, Xi’an, 710054 China; 2 School of Engineering, Newcastle University, Newcastle upon Tyne, NE1 7RU UK; 3 Key Laboratory of Western China’s Mineral Resources and Geological Engineering, Ministry of Education, Xi’an, 710054 China; 4 Department of Earth and Environmental Sciences, Università degli Studi di Milano Bicocca, Piazza della Scienza 4, Milan, 20126 Italy

**Keywords:** Natural hazards, Seismology

## Abstract

Earthquake triggered landslides often pose a great threat to human life and property. Emerging research has been devoted to documenting coseismic landslides failed during or shortly after earthquakes, however, the long-term seismic effect that causes unstable landslides only to accelerate, moderately or acutely, without immediate failures is largely neglected. Here we show the activation and recovery of these earthquake accelerated landslides (EALs) in Central Italy, based on satellite radar observations. Unlike previous studies based on single or discrete landslides, we established a large inventory of 819 EALs and statistically quantified their spatial clustering features against a set of conditioning factors, thus finding that EALs did not rely on strong seismic shaking or hanging wall effects to occur and larger landslides were more likely to accelerate after earthquakes than smaller ones. We also discovered their accelerating-to-recovering sliding dynamics, and how they differed from the collapsed 759 coseismic landslides. These findings contribute to a more comprehensive understanding of the earthquake-triggering landslide mechanism and are of great significance for long-term landslide risk assessment in seismically active areas.

## Abstract (web summary)

This study uses satellite radar observations to investigate the triggering and recovery mechanisms of landslides that are accelerated by earthquakes without immediate failures but showing a prolonged response.

## Introduction

Landslides refer to mass wasting on the ground surface, causing severe casualties and economic losses each year either instantaneously from rapid slope failures^1^ or accumulatively from slow-to-fast downslope movements of soil and/or rocks^2^. The slope instability of a landslide can be triggered by earthquakes^3–5^, rainfall^6^, snowmelt^7^, volcanic activities^8^ and disturbances from anthropogenic activities^9^. Among them, the Earthquake Triggered Landslides (ETLs), occurring immediately following an earthquake^10^ or after a period of time^11^, accounted for over 60% of landslide casualties between 2002 and 2010^12^ and are a major concern, especially in seismic active regions. This has motivated plentiful studies with a focus on coseismic landslides that collapsed during or within a short period (seconds to minutes) after earthquakes^10,13,14^, new post-seismic landslides that developed into failures under the action of aftershocks or post-seismic rainfalls along earthquake-cracked slopes^15,16^, and post-seismic reactivations/remobilizations of coseismic landslide deposits that occurred mostly during rainfall events^17^. However, long-term seismic effects that activate unstable landslides but without causing failures/collapse, even after a long period since the earthquake (months to years), are typically ignored due to minor, if any, ground changes caused compared to collapsed slopes. These landslides (referred to as Earthquake Accelerated Landslides, EALs) respond to coseismic or post-seismic stress disturbances differently from the coseismic landslides and other types of collapsed/cracked post-seismic landslides and are typically activated with considerably increased displacement velocities compared to their pre-earthquake levels. As a result, EALs may generate continuous damage to the ground or man-made infrastructure above them and develop into catastrophic failures in the future.

Preliminary attempts have located a single EAL^18,19^ or limited neighboring EALs^12^. For example, Bontemps et al.^18^ used 3-year geodetic and seismic datasets to characterize a slow-moving landslide affected by local earthquakes and seasonal rainfall, and highlighted how small-shaking events weakened the landslide rigidity. Lacroix et al.^12^ detected nine slow-moving landslides in the Colca valley (Peru) with Pléiades images and reported their accelerations were caused by a regional Mw 6.0 earthquake. However, due to the lack of a complete and consistent EAL inventory after earthquakes, these localized studies only characterized individual EALs and were unable to investigate collectively the landslide behaviors in the perspective of an integral EAL inventory. The spatial-temporal features of EALs such as the spatial pattern of landslide distribution, the different behaviors between EALs and coseismic landslides, and their overall evolution of the sliding velocity were largely unknown. These features may well explain the landslide-triggering mechanisms and contribute to hazard early warning or prediction.

In this context, establishing a complete EAL inventory consistently over a sufficiently large spatial extent and a long period becomes essential. At such a spatial-temporal coverage, various potential landslide conditioning factors (LCFs, e.g., seismic effects, slope, lithology) can be related statistically against the EAL occurrence and different temporal behaviors of EALs before and after the earthquake can be distinguished. In this study, we employed a novel InSAR-based EAL detection method to establish an EAL inventory of the 2016-2017 Central Italy earthquake sequence using six years of Sentinel-1 data in both descending and ascending modes from 2014 to 2020. By comparing the identified EALs with the landslides not accelerated by earthquakes (non-EALs) established using InSAR and the Italian national landslide inventory (IFFI^20^), we investigated 15 LCFs and quantitatively classified their impacts on landslide acceleration based on the Information Gain (IG) function^21^. We further investigated the different spatial patterns between EALs and coseismic landslides and the different temporal behaviors of EALs before and after the earthquakes using high temporal resolution InSAR time series. These investigations provide a more complete picture of the landslide triggering mechanisms in addition to the extensively studied coseismic landslides and contribute to a more comprehensive long-term assessment of landslide risk.

## Results

### The 2016-2017 Central Italy earthquake sequence

**Fig. 1:**
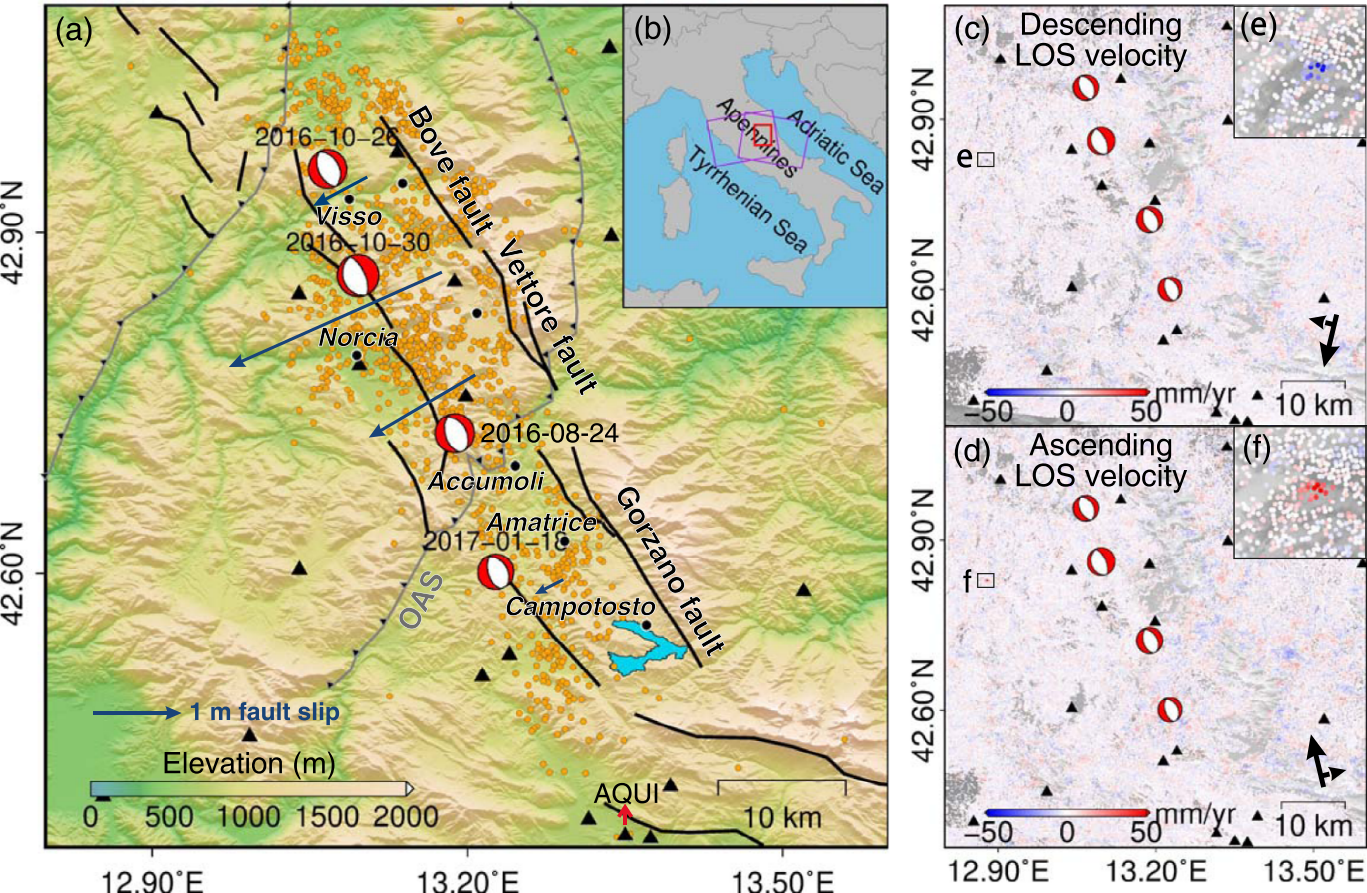
Seismotectonic background of the study area and velocity fields along the radar Line of Sight (LOS) direction derived from Interferometric Synthetic Aperture Radar (InSAR). **a** Seismotectonic background with the four 2016–2017 earthquakes. Solid black lines represent the major active faults while gray barbed lines indicate the pre-existing compressional faults^22^. The locations and moment tensor solutions of the four main earthquakes in Central Italy from 2016 to 2017 (red beach balls) were obtained from the United States Geological Survey (USGS). Dark blue arrows indicate the maximum fault slips of the four events^22,74^. Orange dots represent aftershocks (M > 3.0). Black triangles mark Global Positioning System (GPS) stations, and black solid circles represent major cities. **b** Geographical location of the study area (red rectangle). Purple rectangles indicate the coverage of descending- and ascending-track Sentinel-1 images. **c** and **d** are the filtered post-earthquake descending and ascending LOS velocity fields, with positive values implying the Earth’s surface moving away from the satellite. The insets (**e**) and (**f**) are exampled zoom-in views of the InSAR velocity.

The study area, Central Italy, accommodates an influential earthquake sequence including four main events that occurred respectively on 24 August 2016 (Mw 6.1), 26 October 2016 (Mw 5.9), 30 October 2016 (Mw 6.6) and 18 January 2017 (Mw 5.5), which struck a wide area of Central Apennines (Fig. 1a). The four events caused more than 300 casualties and severely damaged buildings and transportation routes^14^. The earthquake sequence mainly ruptured the Mt Gorzano-Vettore-Bove (MGVB) fault system trending NW-SE, with normal fault slipping^22^. According to the geodetic inversion and the relocation of aftershocks, an antithetic NE dipping normal fault near the Norcia area was additionally discovered to be ruptured during the 30 October 2016 event^23,24^. The slip state of another inherited west-dipping thrust, the Olevano‐Antrodoco‐Sibillini (OAS) thrust, was also widely discussed^24–26^ but its role in the rupture geometry and the reactivation mechanism remained unclear^23^. In addition to the coseismic ruptures, centimeter-level post-seismic surface deformation following a logarithmic temporal decay was also observed and the related shallow afterslip was revealed to likely halt the rupture propagation^27^. Such complex seismotectonic background poses a challenge to large-scale EAL detection and prompts us to develop a new EAL detection method based on the InSAR-derived velocity fields (Fig. 1c, d) as described in the Methods section.

### Spatial distribution of earthquake-accelerated landslides

**Fig. 2:**
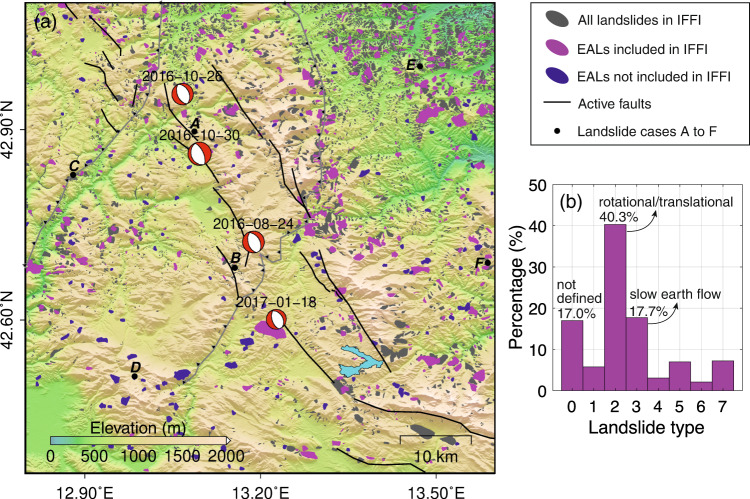
Established Earthquake Accelerated Landslide (EAL) inventory. **a** Distribution of detected EALs and landslides documented by the inventory of landslide phenomena in Italy (IFFI). Gray polygons are IFFI-documented landslides. Magenta and navy-blue polygons are respectively EALs already in IFFI and EALs not in IFFI. Black dots indicate the spatial locations of six landslide cases used for temporal analysis. **b** Proportion of different types of EALs. Note that these landslide types are from IFFI’s historical records with the following codes. 0: not defined, 1: fall/topple, 2: rotational/translational, 3: slow earth flow, 4: rapid debris flow, 5: complex, 6: Deep Seated Gravitational Slope Deformations (DSGSD), 7: shallow landslides.

The InSAR-derived EAL inventory reveals a wide-spreading distribution of EALs in the study area. Compared with the existing IFFI (partly updated to 2007 and partly to 2017), we detected a total of 819 EALs, among which 684 (83.5%, magenta polygons in Fig. 2a) were already documented in IFFI and the remaining 135 (16.5%, navy-blue polygons in Fig. 2a) were newly detected landslides. Not all the landslides in IFFI were detected by InSAR as they were either not moving or not accelerated by the earthquakes. Note that the final boundaries of EALs determined by InSAR may not be entirely consistent with IFFI due to their different temporal coverages (detailed in Methods). As shown in Supplementary Fig. 1, the newly detected landslides by InSAR are mainly distributed on the southwest side (Lazio Region) of the seismogenic fault, which could be due to the low density of the documented landslides in this area (the IFFI was only updated to 2007). We also noticed that there is no significant relation between the occurrence of new EALs and the distance to the seismogenic fault as new EALs can occur in areas either near or far from the MGVB fault system. According to IFFI, 40.3% of the total 819 InSAR-detected EALs are rotational/translational landslides and 17.7% are slow earth flows (Fig. 2b). These two types of landslides are also dominant in the whole IFFI inventory within the study area. There are 17.0% EALs whose types are unclear, including four previously undefined landslides in IFFI and 135 newly detected landslides by InSAR. Note that we cannot rule out the possibility that the detected moving slopes are not authentic landslides, but as the vast majority of EALs (83.5%) are IFFI-verified landslides, we consider the proportion of false detection to be low and will not alter our conclusion.

### Conditioning factors of landslide occurrence

We investigated 15 LCFs (Supplementary Table 1) that may directly trigger EALs or have impacts on the occurrence of EALs. We quantified their impacts according to the IG values (Supplementary Fig. 2a), which represent the influence of a particular LCF on the occurrence of EALs. The greater the IG, the stronger the relationship between the LCF and EAL and the more important the LCF is to the EAL occurrence. We classified the IG values of the 15 LCFs into three categories: prominent, moderate and negligible impacts. Among all the LCFs, the size of the landslide body exerted the strongest effect (Supplementary Fig. 2a). Non-EALs tend to concentrate on smaller landslides with 70.2% being smaller than 0.3 km^2^ compared to 30.8% for EALs as shown in Supplementary Fig. 2b. The proportion difference of landslide numbers between EALs and non-EALs (Supplementary Fig. 2c) more clearly shows EALs contains a greater proportion of larger landslides than non-EALs, implying that larger landslides are more likely to accelerate after the earthquakes than smaller ones. Note that very small EALs and non-EALs (<${9\cdot {10}^{-4}{km}}^{2}$) are not detectable in our case and some adjacent small landslides without clear boundaries could be joined together. This is due to the spatial clustering effect of the InSAR-based (30 m spatial resolution) automated landslide detection method and the principle that an EAL or non-EAL needs to contain at least three InSAR pixels to ensure measurement reliability (see the Methods section). The remarkable effect of landslide size revealed here is consistent with previous studies of failed coseismic and post-seismic landslides^15,17^, which found that larger coseismic landslides were more susceptible to remobilization after earthquakes than smaller ones and also tended to remain active for longer. Although EALs and the post-seismic remobilizations in coseismic deposits are two different types of ETLs, the underlying activation mechanisms may be similar, such as a high proportion of weak materials in large landslides, leading to a decrease in the frictional strength with increasing landslide size, as also observed in other studies^28,29^. Therefore, the landslide size could be a generic conditioning factor of landslide susceptibility after earthquakes, especially in the far field, where the faster attenuation of high-frequency waves with respect to low frequency may cause a slope resonance that favors larger landslides with respect to smaller ones^30^. While this effect of resonance is completely obscured by the effect of high shaking intensity for collapsed coseismic landslides close to the epicenter^4^, it is particularly important for those far-field EALs as the seismic induced stress changes may be already weak in the far field. This will be investigated further in the discussion section.

The second most influential factor is lithology. As shown in Supplementary Fig. 2d, the top three landslide compositions in the study area are sandstones/claystones (with limestones and evaporites), marl, and limestones/marly limestones. There is a larger proportion of sandstone/claystone type landslides among EALs than non-EALs (40% compared to 32%), suggesting this type of landslide has a weak resistance to the post-earthquake acceleration effect. The third most influential factor is the pre-earthquake landslide activity indicator, i.e., the pre-earthquake velocity. Surprisingly, its distribution histogram (Supplementary Fig. 2e) does not lean towards the high-velocity side, which reveals that most EALs were not highly active before the earthquakes. In contrast, active landslides with pre-earthquake velocities greater than 12 mm/yr seemed to have been less affected by the earthquakes. Possible explanations could be: (i) the force induced by earthquakes was insignificant compared to their original driving factors (e.g., gravity and rainfall) and was hence not strong enough to alter their state of motion; or (ii) they might have accelerated shortly after the earthquakes and then quickly decelerated back to their original state due to the rate- and state-dependent frictional properties^19,31^. Similar landslide behaviors were observed in the Trishuli River catchment, Nepal, where 6 slow-moving landslides with velocities greater than 20 mm/yr driven by monsoonal precipitation were not accelerated by the 2015 Mw 7.8 Nepal earthquake^32^.

Among all the topographic LCFs, the positive openness (representing the surface convexity) has a prominent influence on EALs, and the slope and aspect angles, commonly used topographic variables in assessing landslide hazards, have moderate influence according to the IG ranking. EALs were found to be more prone to have high positive openness (~80, Supplementary Fig. 2f) than non-EALs. The histogram of slope angles (Supplementary Fig. 3a) shows that the preferred slope angle of EALs was between 10 and 20 degrees and the rose diagram of aspect angles (Supplementary Fig. 3b) shows that the orientations of EALs were concentrated between SE and SSW directions. In addition, compared to non-EALs, EALs also exhibited an increased proportion in the NNW and NNE directions. These EAL aspect directions are roughly parallel to the strike of the seismogenic faults (SSE-NNW, solid black line in Supplementary Fig. 3b) and perpendicular to the direction of normal fault slips, suggesting the existence of a directional effect. However, the cause of this effect is inconclusive. Some studies^5,13,33^ argued that landslides with a slope aspect parallel to the fault slip direction are more susceptible to failure during earthquakes, but there are other studies^10,34^ reporting the prevalent landslide orientation to be normal to the fault ruptures. The difference is that almost all previous studies were focused on coseismic landslides that collapsed during earthquakes whilst we focused primarily on EALs, which may represent a different spatial pattern as will be shown later.

**Fig. 3:**
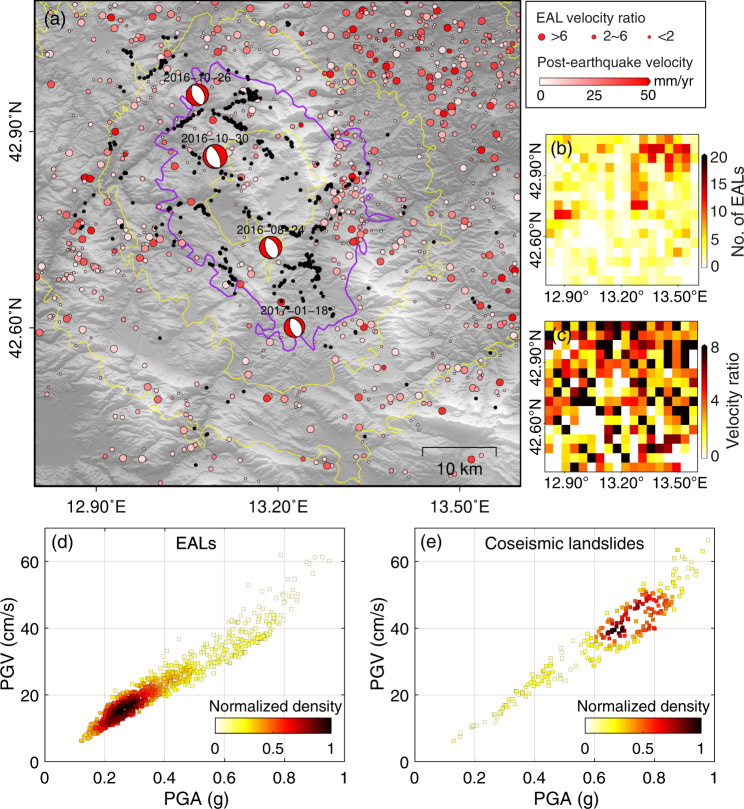
Distribution of Earthquake Accelerated Landslides (EALs) and coseismic landslides and their relationships with Peak Ground Acceleration/Velocity (PGA/PGV). **a** Postearthquake deformation velocity of EALs and the EAL velocity ratio. Solid lines indicate the cumulative PGA contours of the four earthquakes, where the purple contour shows 0.6 g PGA. Black dots are coseismic landslides documented by Martino et al. (2019)^14^. **b** Number of EALs on a resampled grid with a cell size of 5 km × 5 km. **c** Average velocity ratio of EALs on the grid. **d** and **e** show the density scatter plots respectively of EALs and coseismic landslides against PGA/PGV.

The IG values of Peak Ground Velocity (PGV) and Acceleration (PGA) are moderate and rank in the middle (7-8) of all LCFs. To investigate the detailed spatial relationship between seismic ground shaking and EALs, we displayed the PGA counters, the earthquake epicenters, and the landslide velocity ratio before and after the earthquakes in Fig. 3a. Unlike the coseismic landslides (collapsed shortly after the mainshock, black dots in Fig. 3a, a total of 759^14^) which were distributed mostly near the epicenters, EALs had a wide distribution and were not concentrating inside the high PGA area (e.g., inside the purple PGA contour in Fig. 3a). We further plotted in Fig. 3d, e their density scatters against PGA and PGV (each dot represents the landslide density under the corresponding PGA/PGV), in which an opposite correlation was observed between these two types of ETLs. Coseismic landslides tend to appear in areas with strong shaking, whilst EALs tend to occur in light-to-moderate ground-shaking areas. We also constructed a uniform grid map within the study area with a cell size of 5 km × 5 km, and for each grid, we counted the number of EALs (Fig. 3b) with the ratio between their average velocities before and after the earthquakes (i.e., the velocity ratio shown in Fig. 3c). More EALs were distributed in the northeast which is consistent with the distribution of landslides in IFFI (Fig. 2a) but the distribution of the EAL velocity ratio was almost uniform in space. It is asserted that weak ground shaking far away from the epicenter was enough to cause notable accelerations to the landslide movement and greater ground shaking did not necessarily mean the larger potential to accelerate landslides or larger accelerations. On the one hand, most unstable landslides near the epicenter had collapsed during the mainshock, leaving most EALs identified in the far field. On the other hand, the landslide rigidity could be altered by relatively weak ground shaking and its kinematic behavior may not directly be related to the magnitude of ground shaking. For example, Bontemps et al.^18^ observed diverse responses of a slow-moving landslide in Peru to a series of small-to-medium earthquakes (Ml < 4.5). Lacroix et al.^19^ found the post-seismic motion of a landslide triggered by an Mw 6.0 earthquake 20 km away was even 3 times larger than the coseismic displacement.

### Post-earthquake dynamics of earthquake-accelerated landslides

Apart from detection and spatial analysis of distributed EALs using mean velocity maps, InSAR-derived deformation time series can reveal the temporal evolution characteristics of EALs at time intervals of 6 to 12 days. We started by looking at individual EALs and plotted the sliding velocities of six examples in Supplementary Figs. 4 and 5. Note that the linear sliding velocity (Supplementary Fig. 4) is based on the assumption that landslides mainly move along the slope direction^35^, which may not be suitable for all InSAR pixels but is sufficient to reveal the scale and evolution of the underlying movement. The velocity fields verify the reliability of our landslide detection results as most InSAR pixels are distributed within uniform slope cells without noisy pixels and apparent slope cell aliasing. They also show that the active parts of landslides are well outlined, which ensures the restoration of the most significant movement features. The epoch-by-epoch velocity of each EAL (Supplementary Fig. 5) was computed by linearly fitting the InSAR LOS displacement time series within a fixed 3-month time window and projected onto the slope direction using Eq. (3) in the Methods section. The six EALs were distributed on both sides of the seismogenic faults (locations shown in Fig. 2a) with a distance of <10 km (A and B, on the hanging wall of the faults), 10 to 25 km (E and F, footwall) and >25 km (C and D, hanging wall), respectively. All the six EALs have considerable velocity ratios (>2.0) regardless of the distance to the fault, and, as noticed in Fig. 3c, being closer to the fault does not necessarily imply larger velocity changes. For example, the closest EAL A only showed a moderate velocity ratio (2.5), whilst EALs B and C, farther than A, had larger ratios (6.6 and 4.6) and EAL D (farthest from the fault, 32.2 km) exhibited the largest ratio (23.3). EALs E and F that were distributed on the footwall have even larger sliding velocities than the EALs (A, B and D) on the hanging wall which is generally considered to receive more seismic energy, with stronger ground motion in dip-slip events^36,37^. This suggests the hanging wall effect on the EAL velocity is marginal, probably because the EAL does not depend on strong coseismic ground motions, as analyzed in the previous section according to Fig. 3.

One notable dynamic feature shown in Supplementary Fig. 5 is that all the six EALs were dominated by stronger velocity fluctuations after the earthquakes as compared to relatively flat variations before the earthquakes. The variation of the post-earthquake velocity correlated with the precipitation time series (monthly precipitation aggregated from daily Global Precipitation Measurement (GPM) records) and most velocity peaks (red dotted rectangles) were accompanied by local precipitation peaks. We should note that the correspondence is not perfect since the precipitation may not be the only force dominating the activity of these EALs after the earthquakes and other factors (e.g., landslide depth, joints and geomorphological discontinuities, soil moisture and soil strength) could collectively affect the sensitivity of landslides to rainfall/precipitation^38–40^. The complex interactions of these factors may lead to a delayed or heterogeneous response of landslides to rainfall inputs and the relation between the landslide activity and precipitation is thus more sensitive to local effects^16^. Nevertheless, compared to the pre-earthquake level, the response of velocity to precipitation after the earthquakes is clearly much stronger, implying that the accelerated landslides became more susceptible to precipitation than before. Such changes may be caused by the generation of preferential paths for water infiltration in landslide bodies due to soil damage after the earthquake^16,18,41^. The soil damage could be manifested as microfractures^42^, which makes it easier for water to penetrate the landslide body and increases the sensitivity of the landslide body to precipitation. To conclude, the enlarged velocity fluctuations in response to precipitation, together with the post-earthquake sliding acceleration, have collectively weakened the stability of EALs.

**Fig. 4:**
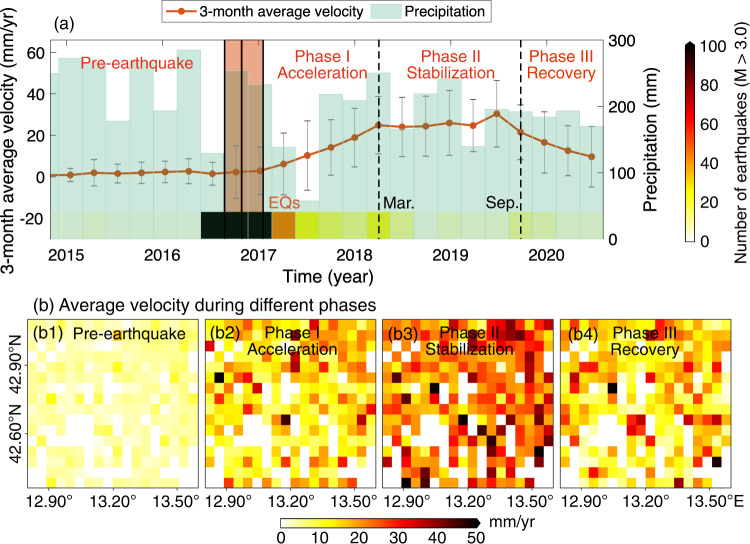
Average velocity of Earthquake Accelerated Landslides (EALs) in the study area estimated every three months. **a** Times series of the 3-month mean velocity and corresponding precipitation. The red shading indicates the period of the 2016-2017 earthquake sequence, and the black vertical solid lines indicate the specific time of the four earthquakes. The colored rectangles at the bottom of (**a**) represent the number of earthquakes (M > 3.0) in their time windows. Note that the gray error bars are the standard deviations of all the EAL velocities and represent the velocity dispersion between EALs rather than a measure of the velocity accuracy. **b** Gridded average velocity of EALs during different phases.

Benefiting from a complete EAL inventory, the overall responding mechanism of EALs within the study area, rather than a single EAL, can be investigated. We calculated the 3-month mean velocity of all the detected EALs and plotted in Fig. 4a their averages together with the averaged GPM precipitation in the study area. The 3-month mean velocity of each EAL was computed in the same way as in Supplementary Fig. 5 and then averaged across the whole study area, with the standard deviation calculated (gray error bars in Fig. 4a, representing the velocity dispersion between EALs). The average velocities before the earthquake sequence remained at a low level close to zero and their standard deviations were smaller compared to their post-earthquake counterparts. After the earthquakes, the velocity began to increase, with each EAL having its own velocity ratio, reflected by the large velocity standard deviation across all the EALs. According to the change of velocities, we identified three distinct velocity evolution phases after the earthquakes and plotted them in Figs. 4b2–4 respectively their velocity distributions using the same method as in Fig. 3b, c but within the correspondent time periods, (1) the acceleration phase, from January 2017 to March 2018, during which a continuous increase in the average velocity was observed, implying a stage where most EALs were experiencing substantial accelerations; (2) the stabilization phase, from March 2018 to September 2019, during which the mean EAL velocity reached a steady state accompanied by a rapid decrease of the number of aftershocks, implying that most EALs had stopped accelerating and were creeping at relatively steady velocities; (3) the recovery phase, from September 2019, during which the average velocity began to decrease, suggesting the effect of the earthquakes was fading away. The fully accelerated velocities were unable to sustain at a high level for long and started to recover (at least partially) ~3 years after the earthquakes.

The phenomenon of landslide recovery was also observed for other earthquakes (e.g., the 1999 Chi-Chi earthquake^43^, the 2008 Wenchuan earthquake^17^ and the 2015 Gorkha earthquake^44^). However, they all focused on new post-seismic landslides or post-seismic reactivations/remobilizations, which are different from EALs with continuous slow-moving dynamics after earthquakes. For the new post-seismic landslide failures or remobilizations, potential mechanisms include the progressive decay of energy from aftershocks, closure of fractures due to the settlement of shaken rock, re-establishment of plant root networks, and erosive removal of debris or weakened materials^36^. But for the EALs, due to the slow-motion feature without slope failures/collapse (i.e., relatively intact landslide bodies), there would be no significant erosive removal of landslide materials after earthquakes and the plant root networks would not suffer from serious seismic damage. The root cohesion is also probably ineffective as a stabilization factor considering the relatively large size of the mapped EALs (see Supplementary Fig. 2c). Instead, the progressive decay of the aftershock energy and the closure of earthquake-generated microfractures are the likely controlling factors for EAL recovery. This can be evidenced by the decreased number of aftershocks in the stabilization and recovery phases as shown in Fig. 4a. The role of the microfracture closure is also justified since the healing process of slow-moving landslides after earthquakes is usually associated with the re-compaction of the soil as microfractures close and grains re-cement together, which reflects a viscoelastic response of the soil^18^. Such a healing process could be considerably slow. For example, the landslide activity after the 2008 Wenchuan earthquake took 10 years to enter the recovery period and required further 15 years to be completely stable^45^. For EALs in this Central Italy earthquake sequence, their recovery seems to be faster. Until August 2020 (4 years after the earthquakes), the average velocity has already shown the trend of returning to the pre-earthquake level. However, there is also a degree of spatial variability in the recovery process, with some of the EALs (i.e., those with large sliding velocities in the grid of Fig. 4b4) recovering more slowly than others. Compared to the pre-earthquake level (Fig. 4b1), the higher spatial variability of the velocity among EALs shown in Fig. 4b4 suggests the existence of longer-term or non-recovering earthquake-induced effects.

## Discussion

In previous sections, we distinguished EALs from coseismic landslides by considering whether they are affected immediately and fail shortly after earthquakes, or can maintain intact for a relatively long period but experience accelerated sliding, which may eventually lead to failure in the future. However, this is not the only difference between them, and it is crucial to investigate in detail their different behaviors to understand the earthquake-related landslide triggering mechanism. This is facilitated by the complete EAL inventory developed in this study and the well-published coseismic landslide inventory^14^.

The first notable difference is their spatial distribution against the earthquake-induced ground motion described by PGA and PGV (Fig. 3). EALs and coseismic landslides tend to respectively occur in areas with light-to-moderate (i.e., in the far field) ground shaking and strong ground shaking (i.e., in the near field). We explain this by considering that the ground-shaking energy generated by the mainshocks is large enough to cause weak and unstable landslides close to the epicenters to collapse shortly after the earthquakes, but the earthquake-induced energy in the far field is relatively weak and gives rise to long low-frequency seismic waves so that the landslides there were only triggered moderately without immediate failures (i.e., becoming EALs). This was also observed by Saroli et al.^46^ who identified a previously unknown paleo-landslide in southern Italy that was triggered by light-to-moderate seismic shaking without a failure but only accelerated sliding. Detailed spatial analysis in Fig. 3c also reveals that the acceleration magnitude has no close correlations with the location of EALs and large accelerations (velocity ratio larger than 4.0) can occur several tens of kilometers away from the epicenter.

We further investigated the rock composition of the landslide body in Supplementary Figs. 2d and 6a which show the dominant lithologies are limestones and clayey limestones for coseismic landslides and sandstones and claystones (with limestones and evaporites) for EALs. This can be explained by the vulnerability of different lithologies. Since clayey limestones are more fragile than sandstone/claystones in nature^47,48^, landslides composed of more clayey limestones are more susceptible to failure during strong seismic ground shaking and develop into coseismic landslides. On the other hand, landslides with finer materials have a longer response time to seismic-induced pore pressure changes and could more easily develop into EALs subject to a delayed but long-lasting post-earthquake effect.

EALs and coseismic landslides also differ in topographic features. We can see from Supplementary Fig. 6b that the preferential positive openness of coseismic landslides is lower than EALs (75 compared to 80), but their slope angles (Supplementary Fig. 6c) tend to be greater than those of EALs, implying the failure of coseismic landslides relied more on steep slopes than EALs. We further visualized the aspect angles of coseismic landslides in Supplementary Fig. 6d in comparison with Supplementary Fig. 3b of EALs. There are fewer coseismic landslides than EALs along the strike direction of the seismogenic faults, suggesting landslides with an aspect close to the strike of the seismogenic faults are more likely to experience acceleration in motion rather than immediate collapse after the earthquakes. However, the type of earthquakes may also play a role that needs further investigation with additionally established EAL inventories of different types of earthquakes.

Overall, this study provides a pioneering spatiotemporal observation of the distributed EALs over large areas, which, unlike previously widely studied coseismic landslides^5,14^, revealed the long-term landslide behaviors in response to earthquakes. Results show that the occurrence of EALs was not dominated by strong seismic shaking or hanging wall effects but was more significantly influenced by landslide size, with large landslides more likely to develop into EALs. We also found a more sensitive response of EALs to precipitation after the earthquakes and three post-earthquake velocity evolution phases of EALs, i.e., the acceleration, stabilization and recovery phases, each with distinctive velocity features. Such phased evolution towards landslide recovery could be controlled by the progressive decay of seismic energy and the closure of earthquake-generated microfractures. Finally, we distinguished the different behaviors between EALs and coseismic landslides in respect of seismic-induced ground shaking, lithology and topographic features.

These findings constitute a more complete picture of earthquake-induced landslide risks, including both coseismic landslide failures and post-seismic landslide dynamics. This gives great implications for hazard monitoring such that immediate and short-term earthquake-induced effects can be investigated by inspecting coseismic landslides in a near-real-time manner to support rapid rescue operations. Then, the investigation of EALs can help to identify potential hazards in medium-to-long terms so that the local community can be provided guidance to quantitatively assess future slope failures and their impacts on lives and properties. Such comprehensive, long-term and continuous investigation of landslide susceptibility will greatly benefit landslide hazard prevention and mitigation. In the investigation of EALs, this study also proposed potential landslide mechanisms to explain the observed phenomena. A more in-depth understanding of the exact role of each mechanism will be possible in the future for individual landslides or groups of landslides of the same type within a relatively homogeneous geological and morphological setting, with the support of evidence from field investigations and geotechnical data. Besides, how frequent earthquakes of different types affect the behavior and stability of existing pre-earthquake landslides (historical ones) is yet to be studied. These questions would be answered in future ETL studies of more earthquake cases in different areas, with both coseismic and post-seismic landslides (especially the easily overlooked EALs) investigated.

## Methods

### InSAR data and processing

Sentinel-1 Terrain Observation by Progressive Scans (TOPS)^49^ data in Interferometric Wide (IW) swath mode was used to capture the deformation in our study area. The Sentinel-1 constellation operated by European Space Agency comprises two polar-orbiting satellites (Sentinel-1A and 1B) performing C-band SAR imaging and offers wide-area monitoring with a minimum 6-day revisit cycle. The spatial resolution of Sentinel-1 acquisitions is about 5 m in range and 20 m in azimuth. We collected 280 Sentinel-1 images in the descending track (Path 22) spanning from 7 October 2014 to 30 August 2020 and 292 images in the ascending track (Path 117) from 13 October 2014 to 30 August 2020. Each SAR image was connected to at least 10 nearest images in time to generate interferometric pairs. Considering that a long temporal baseline could cause strong decorrelation, we excluded interferograms with a temporal baseline greater than three months and finally obtained 1,420 and 1,507 interferometric pairs (Supplementary Fig. 7) for the descending and ascending tracks, respectively.

A time series InSAR processing flow^50^ considering tropospheric delays was used to process the Sentinel-1 data. To generate interferograms, a 30 m Digital Elevation Model (DEM) from the Shuttle Radar Topography Mission (SRTM)^51^ was used to remove topographic phases and geocode interferograms. Tropospheric delay corrections from Generic Atmospheric Correction Online Service (GACOS) for InSAR^52–54^ were applied to each interferogram to reduce the atmospheric effect. These corrected interferograms were then processed by the Small BAseline Subset (SBAS) mode of the Stanford Method for Persistent Scatterers (StaMPS) software^55^ to generate InSAR time series. During the time series processing, the spatial reference was set as the mean phase value in the study area, and InSAR coherent pixels after the phase correction of spatially uncorrelated noise were sampled at 30 m resolution for 3D unwrapping^56^ to improve the processing efficiency. Such SBAS method does not require a pre-defined deformation model to constrain time series and has been proved to be effective in retrieving the coseismic and post-seismic displacements^27,57^.

The resultant InSAR time series was then validated by Global Positioning System (GPS) displacements from 19 stations (Fig. 1) in the Istituto Nazionale di Geofisica e Vulcanologia (INGV) network. GPS time series solutions provided by the Nevada Geodetic Laboratory (NGL)^58^ were projected onto the radar Line of Sight (LOS) direction, following Eq. (1):

1 $${LOS}={\left[\begin{array}{c}-{\sin }{\theta }_{{inc}}{\sin }{\alpha }_{{head}}\\ {\sin }{\theta }_{{inc}}{\cos }{\alpha }_{{head}}\\ -{\cos }{\theta }_{{inc}}\end{array}\right]}^{T}\cdot \left[\begin{array}{c}N\\ E\\ U\end{array}\right]$$

where $N$, $E$ and $U$ are GPS displacements in the North, East and Vertical (Up) directions; ${\theta }_{{inc}}$ is the incidence angle of satellite radar and ${\alpha }_{{head}}$ is the heading angle; ${LOS}$ is the projected displacement along LOS. Then GPS LOS displacement time series were resampled to the SAR acquisition dates. As InSAR observations are relative measurements with a spatial reference, the GPS time series was referenced to AQUI located in Coppito, Province of L’Aquila, and the InSAR reference point was set to the location of AQUI (marked in Fig. 1a). The reason for choosing AQUI is that it is relatively less affected by the coseismic deformation (<5 mm) and has recorded the most complete GPS data in the past 10 years without interruption. We compared the InSAR and GPS displacements for each observation epoch before and after the earthquake sequence. The comparison results (Supplementary Fig. 8) show that the root mean square (RMS) of the differences between GPS and Sentinel-1 InSAR displacements in descending and ascending modes are 6.2 mm and 7.0 mm, respectively. The linear fit between GPS and InSAR LOS displacements and their Pearson’s linear correlations are 0.75 and 0.89 for descending and ascending LOS, respectively. The high correlation with GPS and the small RMS difference implies the reliability of InSAR observations.

### Pre- and post-earthquake InSAR velocity fields

Based on the InSAR displacement time series, we calculated LOS velocity fields respectively for periods before the first event (i.e., pre-earthquake velocity (${v}_{{pre}}^{{LOS}}$) from 13 October 2014 to 21 August 2016) and one year after the last event (i.e., post-earthquake velocity (${v}_{{post}}^{{LOS}}$) from 24 January 2017 to 25 January 2018). Only one year of the displacement time series after the last event was included to highlight the direct acceleration effect due to the earthquakes, which may fade away over time, and to avoid possible velocity variations caused by non-seismic forcings (e.g., heavy rainfall) as noticed in Supplementary Fig. 5. The velocities during these two periods were obtained by linearly fitting the associated displacement time series^59^. Comparing InSAR and co-located GPS-derived LOS velocities, the RMS differences in the ascending and descending LOS were approximately 3.1 and 3.0 mm/yr, respectively.

Landslide motion signals may be contaminated by residual medium-to-long-wavelength deformation (e.g., post-seismic deformation) or errors (atmospheric, orbital and ocean tide loading errors) on InSAR velocity fields. Therefore, to identify localized landslide motions, we first applied a local spatial filter on the velocity fields to reduce the effects of spatially correlated noise (e.g., residual medium-to-long-wavelength errors) and post-seismic deformation, as shown in Supplementary Fig. 9a. Instead of using a fixed global reference point, the local spatial filter referenced the phase of each pixel against the local mean phase averaged within a kernel (i.e., a circular buffering area) surrounding that pixel^32^. We fixed the radius of the kernel to 2 km as suggested by Bekaert et al.^32^ in double-difference phase analysis which minimized the effect of over-filtering on the landslide signals. With this local spatial filter, spatially correlated signals at distances beyond the kernel size can be largely canceled out.

### Italian national landslide inventory

The Inventario dei Fenomeni Franosi (Inventory of Landslide Phenomena) in Italy (IFFI) project, implemented by Istituto Superiore per la Protezione e la Ricerca Ambientale (ISPRA) and regional environmental protection agencies, provides a national landslide database^20^. The landslide inventory was first published online by ISPRA in 2005. Since then, 620,808 landslide sites (www.progettoiffi.isprambiente.it) have been updated by means of satellite images, airborne photos and field investigation. However, only a limited number of landslides in IFFI remain active, for example, in the Piedmont region of Italy, only ~15% of landslides in IFFI were classified as active by InSAR^60^. In addition, different regions differ in the update time of the landslide inventory. Our study area spans four regions, of which the Umbria Region has updated the inventory up to 2017 while the Marche, Lazio and Abruzzo Regions only updated up to 2007. In total, 9,509 landslides shown in Fig. 2a have been documented in the study area, of which 3,615 (38.0% of the inventory) are classified as rotational/translational slides. The second most widely distributed type of landslide is slow earth flow, accounting for 25.7%, followed by shallow landslides (11.3%). Each of the other landslide types, including rapid debris flow (9.5%), rockfalls/topples (9.4%) and complex slides (4.0%), etc., only represents a small percentage of the entire inventory. In our study, we used IFFI to locate non-EALs and compare the spatial characteristics of these non-EALs with EALs to analyze the impact of different LCFs on landslide acceleration.

### Detection method for earthquake-accelerated landslides

The novel EAL detection method we developed includes three main steps: the location of post-earthquake moving pixels, the automatic clustering of moving pixels into landslide bodies, and the identification of EALs from landslide bodies.

Firstly, the post-earthquake InSAR velocity fields (Fig. 1c, d) were used to locate all the moving pixels which may be clustered as active landslides in the follow-on steps (Supplementary Fig. 9a). We used the LIBRA software^61^ to statistically identify the moving pixels based on the Minimum Covariance Determinant (MCD) method^62,63^. The inputs of the software were ${v}_{{post}}^{{LOS}}$ of the InSAR pixels and the outputs were the locations of the identified moving pixels. This method has two notable features: (i) it does not require an empirical velocity threshold and (ii) the moving pixels can be detected adaptively in an automatic way.

Secondly, based on the identified moving pixels, we used the Density-Based Spatial Clustering of Applications with Noise (DBSCAN)^64,65^ algorithm to automatically cluster these pixels into landslide bodies. DBSCAN is a powerful cluster algorithm in statistics but its application in the landslide field is rare. Unlike the commonly used k-means partitioning algorithm, this algorithm is based on the spatial density of pixels without the requirement of a pre-defined number of clusters, which improves the adaptability of clustering. Moreover, the k-means algorithm forcibly clusters all the included pixels and is vulnerable to noise, while the DBSCAN algorithm is able to exclude noisy pixels that lack sufficient connected neighborhoods^64^. DBSCAN defines three types of pixels: core pixels, border pixels and noisy pixels (Supplementary Fig. 9d). A core pixel is located inside a cluster that is surrounded by at least a minimum number (MinPts) of moving pixels within a fixed radius (R). These moving pixels are called the neighborhood moving pixels of the core pixel. In this study, MinPts was set to three to guarantee at least three moving pixels per cluster^60,66^, and R was set to 60 m (twice the InSAR pixel spacing) to connect sufficient pixels. A border pixel is the neighborhood pixel of at least one core pixel, but it has less than MinPts of neighborhood pixels. A noisy pixel is not neighboring to any core pixel. DBSCAN starts with an arbitrary moving pixel $p$: 1) if $p$ is a core pixel, all neighborhood moving pixels of $p$ will be assigned as the same cluster with $p$ and their types (core or border pixel) will be evaluated; 2) repeat step 1) iteratively for all the neighborhood moving pixels of the core and border pixels in the cluster of $p$ until all the moving pixels that should be clustered with $p$ are identified; 3) move to the next moving pixels until all the pixels were clustered.

Among DBSCAN-produced clusters, those with an average slope of less than 3 degrees were masked out because landslides were unlikely to occur on such flat terrain. Note that since we have collected both descending and ascending Sentinel-1 data, two inventories of post-earthquake active landslides will be generated following the above procedure. Thus, we merged them by uniting overlapping landslide bodies and calculated their pre- and postearthquake velocities along the slope (${v}_{{pre}}^{{Slope}}$ and ${v}_{{post}}^{{Slope}}$) by averaging the along-slope velocities of all pixels inside the landslide body. The along-slope velocity ${v}^{{Slope}}$ of each pixel was calculated by projecting the LOS velocity ${v}^{{LOS}}$ onto the landslide slope direction, assuming that the landslide movements occurred along the steepest gradient of the slope^35,60^. The projection can be expressed as Eqs. (2) and (3), where $s$ and $a$ are the slope and aspect angles at the location of InSAR pixels, $\sigma$ and $\alpha$ are the incidence and heading angles of satellite radar, $C$ is the projection coefficient that converts ${v}^{{LOS}}$ to ${v}^{{Slope}}$. It should be noted that $C$ was limited to 0.3 when $0 \, < \, C \, < \, 0.3$ and to −0.3 when $-0.3 \, < \, C \, < \, 0$ to avoid anomalous exaggeration caused by the projection^67^. Besides, for landslides containing both descending- and ascending-mode Sentinel-1 coherent pixels, the along-slope velocities projected from InSAR observations in these two modes were averaged.

2 $$C=-\!{{\cos }}\left(s\right){{\cos }}\left(a\right){{\sin }}\left(\sigma \right){{\sin }}\left(\alpha \right)+{{\cos }}\left(s\right){{\sin }}\left(a\right){{\sin }}\left(\sigma \right){{\cos }}\left(\alpha \right)+{{\sin }}\left(s\right){{\cos }}\left(\sigma \right)$$

3 $${v}^{{Slope}}={v}^{{LOS}}/C$$

Finally, we compared ${v}_{{pre}}^{{Slope}}$ and ${v}_{{post}}^{{Slope}}$of each candidate landslide to identify the accelerated landslides of interest with sufficient velocity changes (${v}_{{post}}^{{Slope}}$/${v}_{{pre}}^{{Slope}}$ ≥ 1.2) and thereby created an EAL inventory. We refer to this ratio as the landslide velocity ratio which represents the tendency of landslides to accelerate or not due to the earthquakes. The active landslides that were not accelerated by the earthquake sequence (${v}_{{post}}^{{Slope}}$/${v}_{{pre}}^{{Slope}}$ < 1.2) were then classified as non-EALs.

Since InSAR can only detect active landslides, the above procedure is unable to locate non-EALs that are dormant both before and after the earthquake sequence. Therefore, as shown in Supplementary Fig. 9b, we used IFFI to find these non-EALs by (1) locating landslides in IFFI that, like EALs, also contain at least three InSAR pixels; (2) projecting ${v}^{{LOS}}$ of pixels in IFFI-landslides to ${v}^{{Slope}}$ with Eqs. (2) and (3); (3) calculating ${v}_{{pre}}^{{Slope}}$ and ${v}_{{post}}^{{Slope}}$ of each IFFI-landslide by averaging ${v}^{{Slope}}$ of pixels inside before and after the earthquakes; 4) identifying dormant landslides whose ${v}_{{pre}}^{{Slope}}$ and ${v}_{{post}}^{{Slope}}$ are both smaller than 5 mm/yr as defined by Cigna et al.^68^. These dormant landslides were then imported to the non-EAL inventory. Finally, in accordance with the above workflow (Supplementary Figs. 9a, b), we created the inventories of both EALs and non-EALs which facilitate the following statistical and spatial analysis.

### Statistical analysis method

Landslide Conditioning Factors (LCFs) are geo-environmental factors that control landslide occurrence, evolvement and potential collapse. Hence, their spatial distribution may play a key role in landslide susceptivity assessment. We used the Information Gain (IG) function to quantitatively rank a set of LCFs linked to EALs in order to statistically investigate the main conditioning factors. IG is one of the fastest and simplest attribute ranking methods^21^ used to select features in a decision tree model^69,70^. The value of IG represents how much contribution of a LCF can affect the EAL occurrence.

As shown in Supplementary Fig. 9c, the first step of implementing the IG method is to create a dataset of LCFs. We selected a large set of LCFs, including topographic, lithologic, vegetation, hydrologic and seismic factors^11^ (Supplementary Table 1). The 30 m SRTM DEM used in InSAR data processing was processed by the SAGA GIS software (http://www.saga-gis.org) to compute topographic factors (e.g., slope, aspect, and curvature). A Sentinel-2 image with almost zero cloud cover (0.2%) on 14 August 2016 was used to calculate Normalized Difference Vegetation Index (NDVI)^71^ based on the near-infrared spectrum (band 8) and red range of the spectrum (band 4). We also collected GPM daily records^72,73^ from multi-satellite gauging to investigate hydro-climatic factors such as rainfall and snowfall. Regarding the seismic effect, Peak Ground Acceleration (PGA) and Peak Ground Velocity (PGV) of the four 2016-2017 earthquakes in Central Italy were extracted from the USGS ShakeMap products (https://earthquake.usgs.gov/data/shakemap). Note that the ground motions of the four earthquakes were accumulated to account for the overall impact of the earthquake sequence. We additionally selected two other types of LCFs to represent the pre-earthquake landslide activity and the size of the landslide body which may also explain the governing mechanisms of EALs. The pre-earthquake landslide activity factors included the pre-earthquake velocity (${v}_{{pre}}^{{Slope}}$) and the proportion of relatively highly active pixels (${v}_{{pre}}^{{Slope}}$> 10 mm/yr) inside a landslide body before the earthquakes. After collecting the LCF dataset, we calculated high-resolution maps for each LCF and resampled them into a 30 m uniform grid as the DEM (i.e., one map per LCF).

To calculate IG of each LCF, we introduced the concept of information entropy^21^, which was used to measure the uncertainty in the classification of the landslide (i.e., whether the landslide is an EAL or non-EAL). We first generated an index map with the same dimension as the LCF maps for the study area, where pixels inside EALs were marked as 1 and pixels inside non-EALs as 0 (Supplementary Fig. 9d). We then randomly selected 1,000,000 pixels on the index map (about 10% of the total) and calculated the information entropy $H({EAL})$ with Eq. (4), where $p\left(i\right)$ is the percentage of the pixels belonging to landslide class $i$ among the total 1,000,000 pixels ($i=1$: EAL; $i=0$: non-EAL); $n$ represents the number of the class (2 in our case).

4 $$H({EAL})=-\mathop{\sum }\limits_{i=1}^{n}p(i){{\log }}_{2}p(i)$$

We then quantified the uncertainty of the landslide class given that the value of a LCF is known using the conditional entropy $H({EAL|LCF})$. As shown in Eq. (5), $l$ represents the value of a LCF, $p\left(l\right)$ denotes the percentage of the sampled pixels whose value is $l$ on the LCF map, and $p\left({i|l}\right)$ represents the percentage of the sampled pixels belonging to landslide class $i$ when its value is $l$ on the LCF map. Thus, with Eq. (5), we computed the conditional entropy of each LCF.

5 $$H({EAL}{{{{{\rm{|}}}}}}{LCF})=-\mathop{\sum}\limits_{l\in {LCF}}p\left(l\right)\mathop{\sum }\limits_{i=1}^{n}p\left(i{{{{{\rm{|}}}}}}l\right){{\log }}_{2}p(i{{{{{\rm{|}}}}}}l)$$

The IG of a LCF is the difference between the information entropy of the landslide class and the conditional entropy of the LCF, as expressed in Eq. (6), representing how much the uncertainty of determining a landslide as EAL has been reduced after knowing the LCF. Following Eqs. (4) to (6), the IG of each LCF can be calculated and ranked to identify the main influencing factors of EALs.

6 $${IG}\left({LCF}\right)=H\left({EAL}\right)-H({EAL}{{{{{\rm{|}}}}}}{LCF})$$

## Supplementary information

Supplementary Information

## Data Availability

The Sentinel-1 and Sentinel-2 data are available from the European Space Agency (ESA) (https://scihub.copernicus.eu/dhus/#/home). The seismicity data are available from the United States Geological Survey (USGS) (https://earthquake.usgs.gov). The historical landslide inventory in Italy (IFFI) is provided by the Istituto Superiore per la Protezione e la Ricerca Ambientale (ISPRA) (https://www.progettoiffi.isprambiente.it). The precipitation data are provided by the NASA/Goddard Space Flight Center’s Mesoscale Atmospheric Processes Laboratory and Precipitation Processing System (PPS), which develop and compute the IMERG as a contribution to GPM, and archived at the NASA GES DISC (https://gpm.nasa.gov/data/directory). The GPS data of the Istituto Nazionale di Geofisica e Vulcanologia (INGV) network are available from the Nevada Geodetic Laboratory (http://geodesy.unr.edu).

## Code availability

The script of the MCD method is included in the LIBRA software, available at https://wis.kuleuven.be/stat/robust/LIBRAfiles/, and the script of the DBSCAN method for InSAR is available at https://github.com/ImagingGeodesy/DBSCAN_InSAR. Other methods of data analysis are illustrated through text or equations and scripts are available from the authors upon request.
