# Supplementary material for: Triggering and recovery of earthquake accelerated landslides in Central Italy revealed by satellite radar observations
Source: Nat Commun. 2022 Nov 29;13:7278. doi: 10.1038/s41467-022-35035-5 (PMC9708834; doi:10.1038/s41467-022-35035-5)
Supplement: Supplementary file 1 — Supplementary Information [file 41467_2022_35035_MOESM1_ESM.pdf]

Supplementary Information for

Triggering and recovery of earthquake accelerated landslides in Central Italy revealed by satellite radar observations

C. Song et al.

This file includes:

Supplementary Figs. 1 to 9

Supplementary Table 1

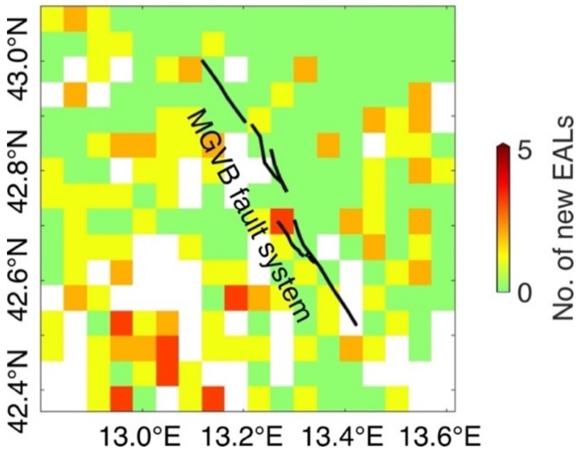

Supplementary Fig. 1 Number of newly detected EALs not included in the IFFI on a resampled grid with a cell size of 5 km × 5 km. Solid black lines represent the Mt Gorzano-Vettore-Bove (MGVB) fault system that includes the major seismogenic faults. The hanging wall is on the west side of the MGVB.

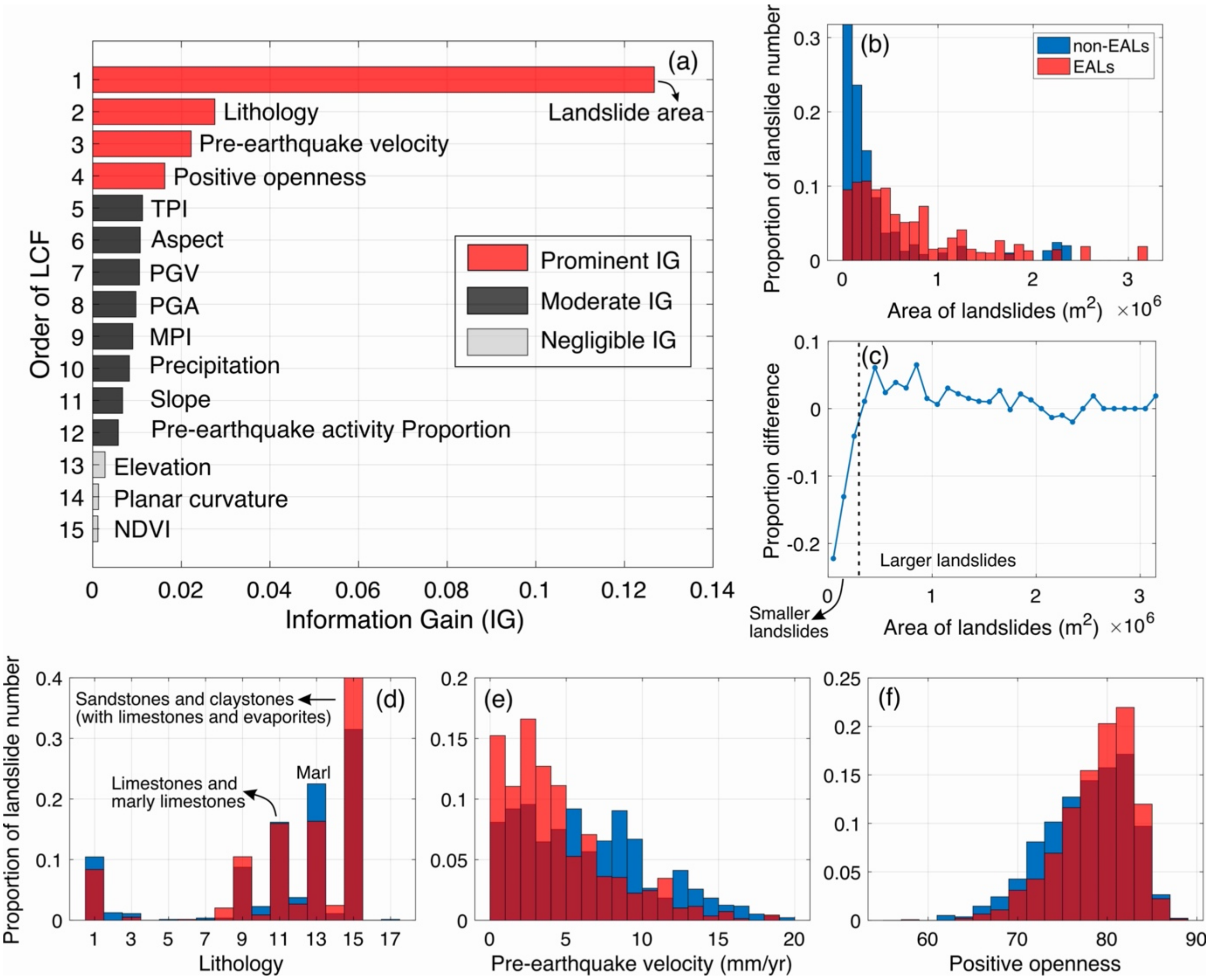

Supplementary Fig. 2 (a) Information gain ranking of the 15 landslide conditioning factors. (b), (d), (e) and (f) are histograms of the four LCFs with prominent IG, i.e., landslide area, lithology, pre-earthquake velocity and positive openness. (c) Proportion difference of landslide number between EALs and non-EALs. In (d), 1: Clays and marls, 2: Dolostones, 3: Fluvial deposits, 4: Glacial deposits, 5: Lacustrine deposits, 6: Lakes and glaciers, 7: Limestones, 8: Limestones and calcarenites, 9:

Limestones and clayey limestones, 10: Limestones and dolostones, 11: Limestones and marly limestones, 12: Limestones, marly limestones and marls, 13: Marls, 14: Sand and conglomerates, 15: Sandstones and claystones (with limestones and evaporites), 16: Sandstones and marly sandstones, 17: Travertine (limestone).

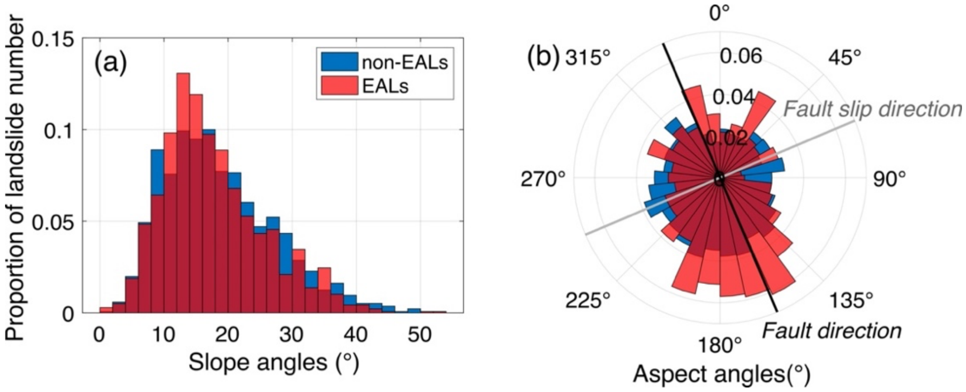

Supplementary Fig. 3 Distribution of slope angles and aspect angles of EALs and non-EALs. (a) Distribution of slope angles. The bin width is set as 2 degrees to include sufficient landslide samples for each bin. (b) Rose diagram of aspect angles. The radial axis represents the proportion of landslide number.

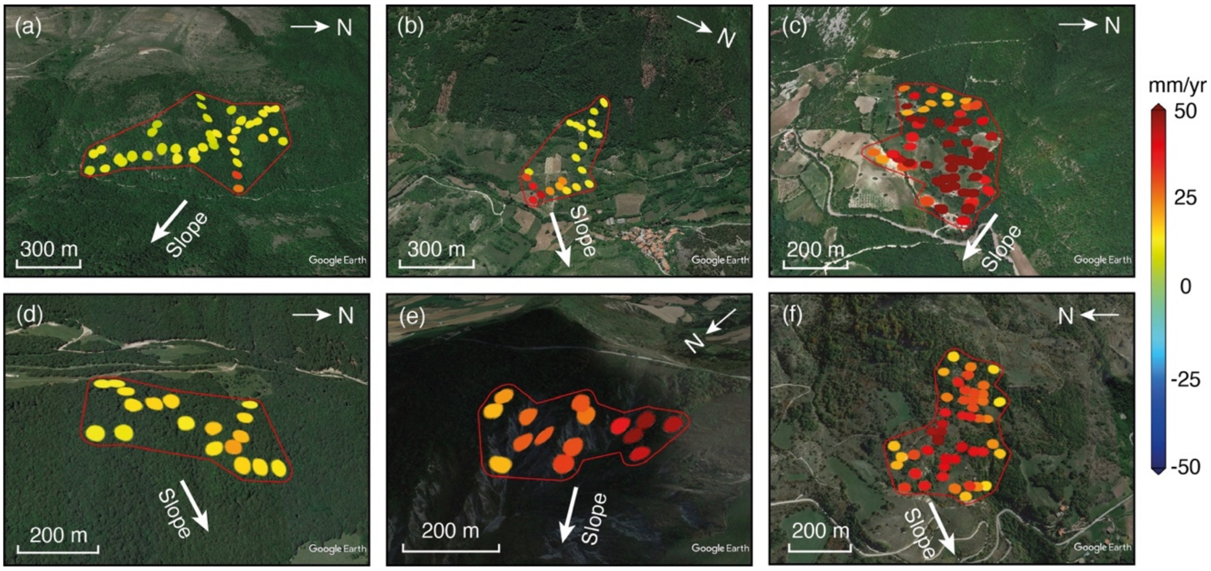

Supplementary Fig. 4 Deformation velocity fields for the six landslide examples. Subplots a to f show EALs A to F respectively (locations shown in Fig. 2). The white arrows represent the average slope directions.

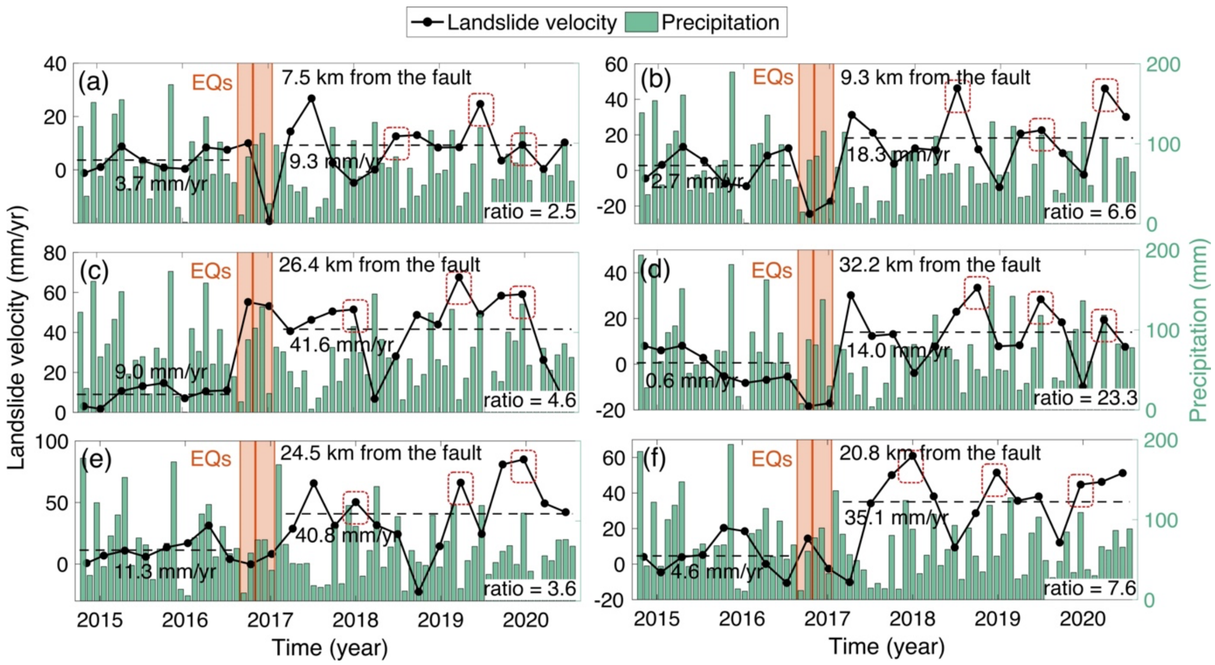

Supplementary Fig. 5 Sliding velocity estimated every three months and the monthly precipitation of the six landslide cases. (a) to (f) represent landslides A to F, respectively. Black dashed lines indicate the mean pre- or post-earthquake velocities, and the velocity ratio is marked at the bottom right of each sub-figure. Red dotted rectangles indicate the velocity peaks after 2018.

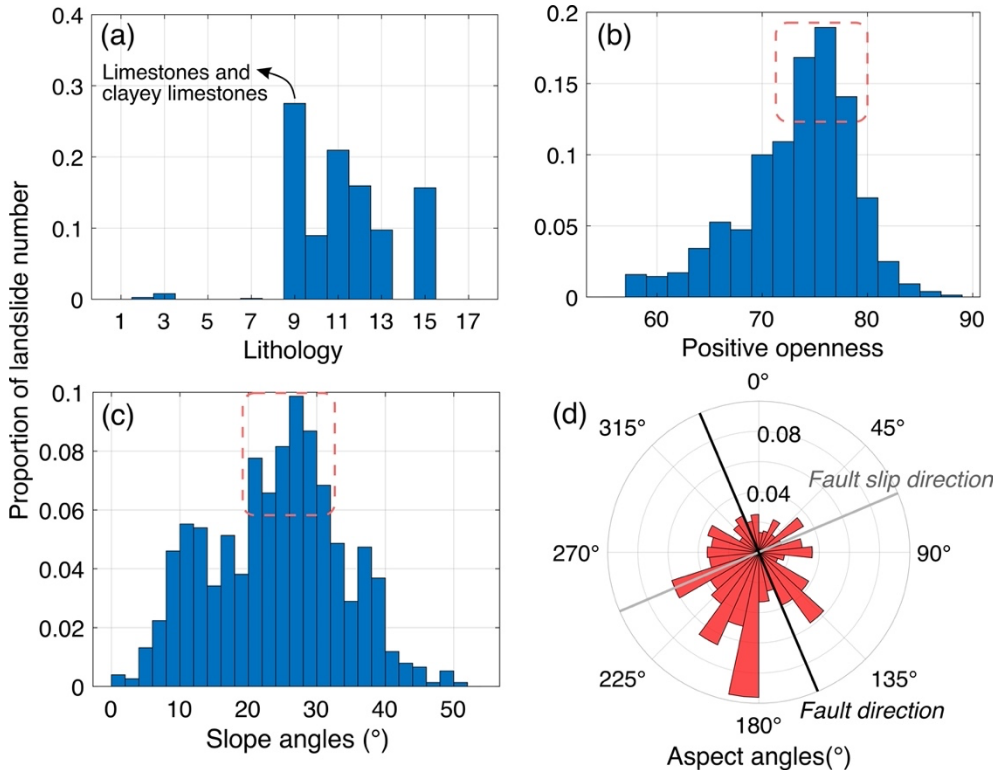

Supplementary Fig. 6 Lithological, and topographic statistics of coseismic landslides. (a) to (c) are histograms of lithology, positive openness and slope angles of coseismic landslides. (d) The rose diagram of aspect angles of coseismic landslides.

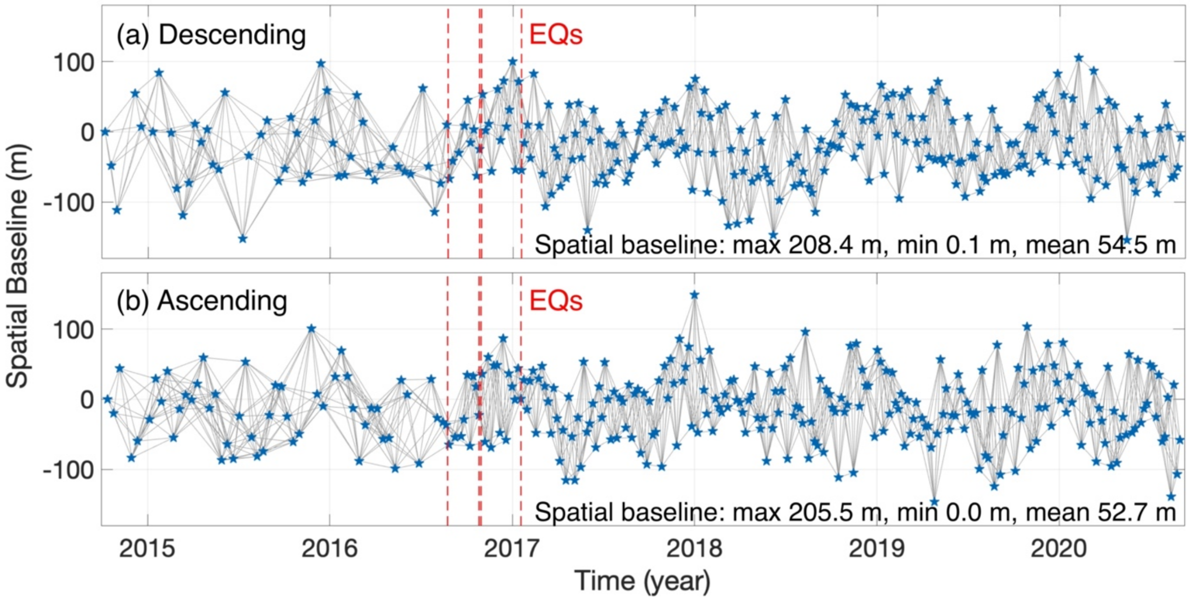

Supplementary Fig. 7 Sentinel-1 acquisition dates and generated interferograms of the (a) descending and (b) ascending tracks. Sentinel-1 acquisitions are indicated by blue stars. The gray lines represent Sentinel-1 interferometric pairs used in the time series analysis and Y-axis shows their perpendicular baseline lengths (m). The red dotted lines indicate the date of earthquakes (EQs).

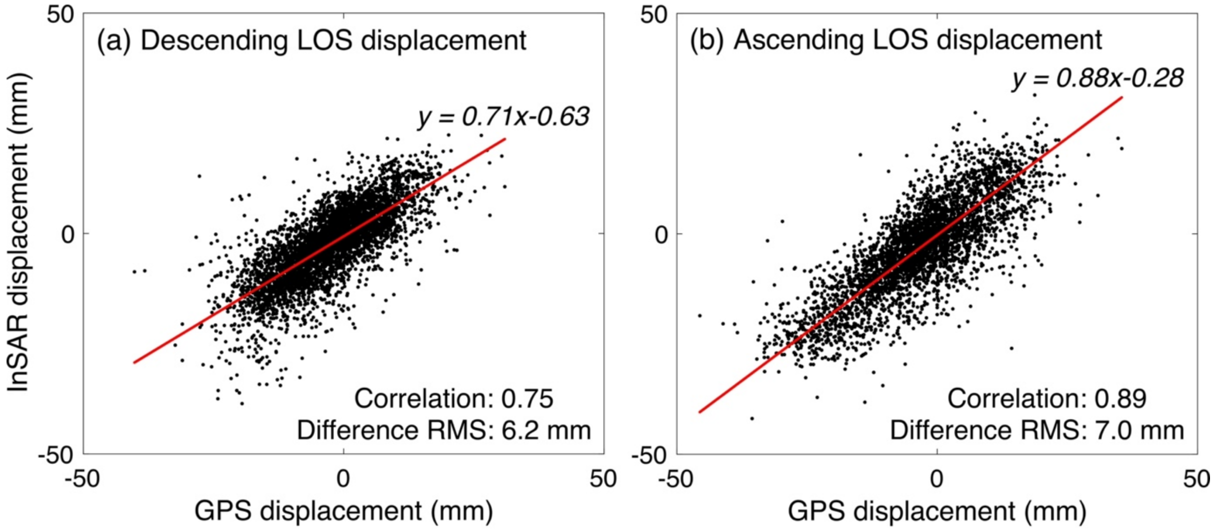

Supplementary Fig. 8 Correlation analyses between InSAR and GPS displacements along LOS for the (a) descending and (b) ascending tracks.

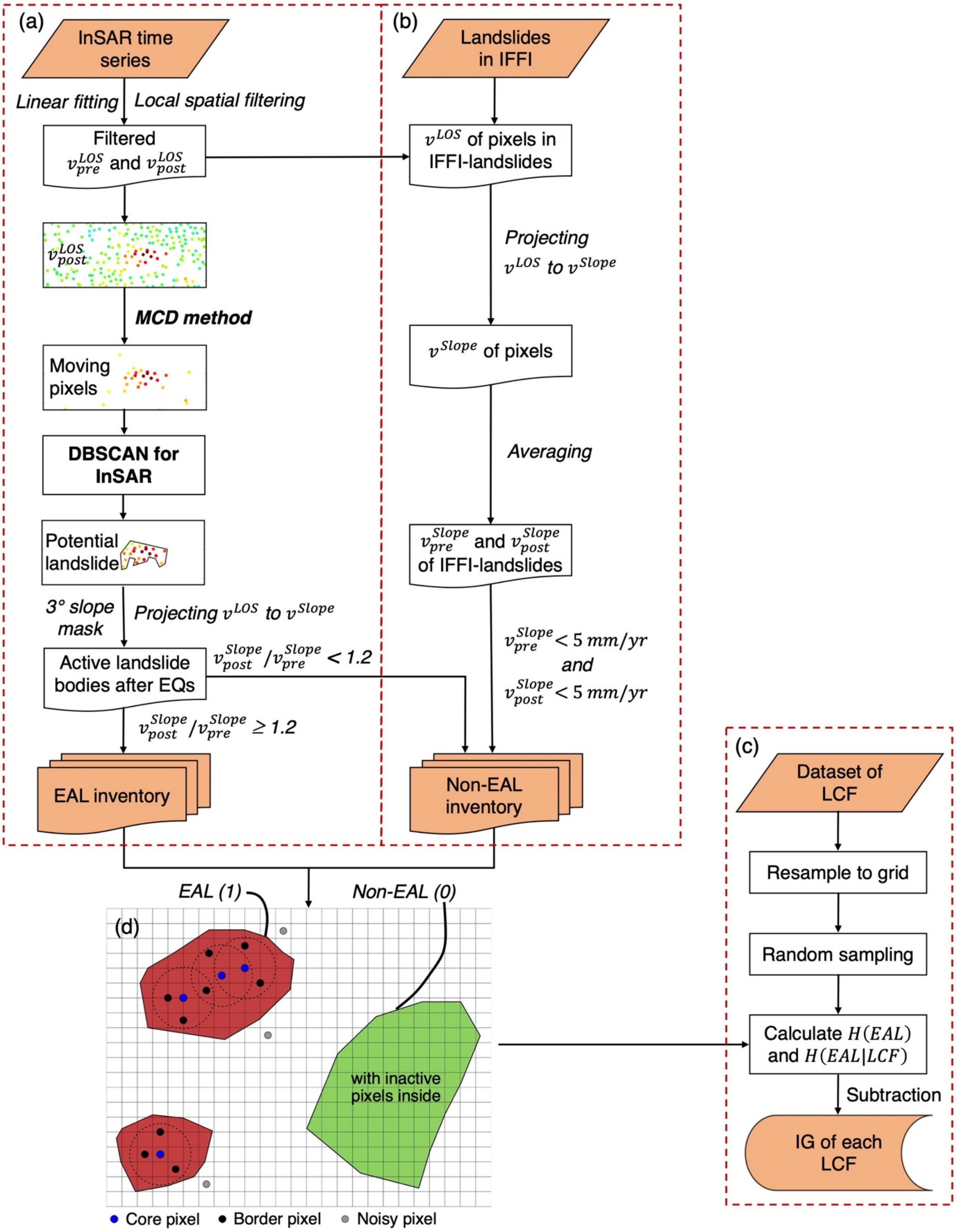

Supplementary Fig. 9 (a) Workflow of developing the EAL inventory based on InSAR. (b) Workflow of detecting non-EALs. (c) Method of Information Gain (IG) for statistically analyzing LCFs. (d) Conceptual diagram of gridding, landslide types and three types of pixels (i.e., core, border, and noisy pixels). Note that both EALs and non-EALs contain at least three InSAR pixels, but for simplicity, only pixels inside the lower left polygon are plotted as an example.

Supplementary Table 1 Landslide conditioning factors used in this study

Type	Landslide conditioning factors	Description
Topographic factors	Elevation	30 m SRTM DEM
	Slope	Slope angles derived from the DEM
	Aspect	Orientation of the slope derived from the DEM, clockwise from the North (0°/360°)
	Planar curvature	Curvature of the slope, relating to the surface shape (>0: convex, <0: concave, 0: linear)
	Positive openness	Surface convexity, indicating how wide a surface can be viewed from any position
	Topographic Position Index (TPI)	Indicating whether the cell position is closer to ridges (>0), valleys (<0) or constant slope (0)
	Morphometric Protection Index (MPI)	Index of how the surrounding relief protects the cell
Lithology	Lithology	Main lithology of the cell
Vegetation	NDVI	Normalized difference vegetation index
Hydro-climatic factors	Precipitation	Gridded 0.1° GPM records during the earthquake sequence, unit: mm
Seismic factors	PGA	Peak ground acceleration, unit: g
	PGV	Peak ground velocity, unit: cm/s
Pre-earthquake landslide activity	Pre-earthquake velocity	Landslide velocity before the earthquakes, unit: mm/yr
	Pre-earthquake activity proportion	Proportion of highly active pixels (>10 mm/yr) inside a landslide before the earthquakes
Landslide size	Landslide area	Unit: m^2
